# The Dietetic Treatment of Zymotic Enteritis, Especially with Regard to the Use of Raw Meat Juice

**Published:** 1900-09

**Authors:** F. Percy Elliott

**Affiliations:** Medical Officer to the Walthamstow Dispensary


### THE DIETETIC TREATMENT OF ZYMOTIC ENTERITIS, ESPECIALLY WITH REGARD TO THE USE OF RAW MEAT JUICE.

BY

F. Percy Elliott, M.B., C.M. Aberd.,

*Medical Officer to the Walthamstow Dispensary.*

It will, I think, be conceded by all familiar with epidemics of zymotic enteritis (so-called summer diarrhœa) that, with the exception of human milk, milk food of any description is, in the majority of cases, badly borne during the early part of the disease. This is especially so when fresh cow's milk (previously boiled) is employed. No matter in what way prepared, whether specially sterilised or peptonised and given weak, it frequently disagrees. Condensed milk, simple or peptonised, will often be found to agree when fresh cow's milk does not; still, in a good number of cases it also is badly tolerated.

At the onset of the disease, it is absolutely necessary to stop giving milk food. Unless milk is completely excluded from the diet, the disease will certainly get worse. When vomiting is severe, food of any kind by mouth should be prohibited for a period of twenty-four to forty-eight hours. It is best to let the stomach have complete rest so far as is possible. If exhaustion is extreme, stimulants will be required and should be given in full doses, administering them *per rectum* if necessary. It is a good plan in those cases where collapse is marked to give an enema composed of two or three teaspoonfuls of stimulant (brandy or whisky) and one or two ounces of beef tea.

When sickness has largely abated, half a teaspoonful of Brand's meat essence, or half to one teaspoonful of a mixture of ten to twenty parts of boiled water and one of Valentine's meat juice, may be given every hour or two by mouth. Bovinine is also a useful preparation here. When any of these are retained satisfactorily, the bulk of food may be gradually increased and given at longer intervals. That food must only be given in small and oft-repeated quantities is a *sine quanon* in treating the disease at this stage.

Where vomiting is not urgent, boiled water or barley water should be given freely, especially if the diarrhœa is excessive, in order to rinse out the gastro-intestinal tract and to supply a part of the fluid lost. Barley water, on account of its soothing effect on the alimentary mucosa, is perhaps preferable to simple boiled water; barley water contains a slight amount of nourishment also, and appeals more to the parents and friends.

Lime water sometimes answers better than either of the above, as from its sedative and astringent properties it is more likely to relieve vomiting and diarrhœa; its power over barley water in this disease is not as great as one would expect however. In older children, thin arrowroot and rice water are also useful. Broths made from mutton, veal, or chicken, with all fatty particles removed, and mixed with boiled water or barley water, form the most satisfactory diet in this stage of the disease. To make it palatable for very young children, the mixture should be sweetened with white sugar, or, better still, milk sugar. Along with this food the meat essences should be used, giving them alternately or adding them to the broth, which should not be hot.

The exclusive use of animal broths is necessarily limited to a short time. To continue using them exclusively is to starve the child, and too long starvation is often a fatal mistake. Meat essences supply a greater amount of nourishment, but this being comparatively small, even their utility is restricted. A change must be made then, if the child is to gain in weight and be contented with its food. In most cases a return to milk food will be accomplished successfully after an interval of two or three days, providing the milk be peptonised and well diluted, and given in small quantities to begin with.

In some of the more severe cases of zymotic enteritis, however, the return to milk food provokes the diarrhœa and increases abdominal pain. Milk food may have to be withheld for several weeks. It is in these cases that raw meat juice proves so serviceable; the writer cannot speak too highly of its value in this connection. It is very nutritious, containing a large amount of proteid, and in virtue of its richness in extractives very stimulating. It is also rich in mineral matter, and possesses valuable antiscorbutic properties. Above all, raw meat juice properly made is easily digested and readily taken by children.

The best way of preparing raw meat juice is to mince up finely raw rump steak and allow it to soak in four times its bulk of *cold* water (the water being always first boiled) for half an hour. The pieces of meat are then squeezed in a piece of muslin and all the juice thus extracted.

There are certain precautions necessary in the preparation and use of raw meat juice. The meat should always be soaked in *cold* water; the juice should be perfectly fresh and above suspicion, therefore it may be necessary to prepare it three or four times daily; after being made, raw meat juice should be kept covered in a cool room to which there is free access of air, and it should never be administered in a hot medium.

Raw meat juice may be given *per se*, a little sugar being added to make it acceptable to a fastidious child. Cautiously prescribed, raw meat juice may entirely replace the proteid in cow's milk. Commencing with a teaspoonful or less, thrice daily or every four hours, it may be increased to two or three ounces in twenty-four hours, according to the age of the child. The best index as to the amount capable of being digested is the stools of the patient. With the addition of cream and sugar, the deficiency in hydrocarbon and carbohydrate is made good; and providing cream can be procured fresh, this may be given with raw meat juice at a comparatively early stage in zymotic enteritis, as it is easily digested in most cases. Owing to its laxative effect, due care must be exercised as to the quantity given at first. Where it cannot be had fresh, or is prohibited for pecuniary reasons, cod-liver oil may be substituted, and this is quite as efficient. If cream prove too laxative, fat may be supplied to the body by rubbing in the cod-liver oil; to disguise its objectionable odour when exhibited in this manner, a little linimentum saponis co. (B.P.) should be mixed with it. The question of supplying fat to the economy in zymotic enteritis does not seriously arise until the child is on the high road to recovery, and at this time it will generally tolerate cream or bear the inunctions of cod-liver oil.

Bread jelly is also a useful food in zymotic enteritis. It is deficient in all the elements required in a food except the carbohydrate, and to obtain a sufficient quantity of this the child would be called upon to consume an amount of bread jelly too large to be dealt with satisfactorily by its digestive organs. Therefore, mixed with water alone, it can only be of service temporarily. However, it is very soothing to the mucous membrane of the alimentary tract, and mixed with raw meat juice and cream in suitable proportions, a little sugar being added, a complete food results. If the jelly is made from bread composed of "seconds" flour, it contains a larger amount of proteid and mineral matter. Bread jelly is prepared by soaking a thick slice of stale bread (two or three days old) without crust in cold water for six or seven hours, so as to get rid of any injurious material that may be present in the bread; at the end of this time the bread is squeezed dry and boiled slowly in a pint of fresh water for a couple of hours. (This enables the process of starch conversion to be more easily accomplished by the digestive organs.) The gruel thus obtained is first strained and next rubbed through muslin or a *fine* sieve and put by to cool, when it soon becomes a jelly. When required for use, a sufficient quantity of the jelly and water (first boiled) are mixed to form a food which will be easily sucked through the bottle; about half an ounce of jelly to eight ounces of water will be the proportions. Bread jelly keeps badly, and the precautions mentioned in the preparation of raw meat juice should be observed here.

In the later stages of zymotic enteritis, and in a great many cases, in the earlier stages, when vomiting has ceased to be troublesome, weak peptonised milk can be added to bread jelly, the dilution being lessened and peptonisation abolished gradually as convalescence proceeds. Fat of course must be supplied to bring the food up to a standard one.

